# The Long Noncoding RNA *MEG3* Contributes to Cisplatin Resistance of Human Lung Adenocarcinoma

**DOI:** 10.1371/journal.pone.0114586

**Published:** 2015-05-20

**Authors:** Jing Liu, Li Wan, Kaihua Lu, Ming Sun, Xuan Pan, Ping Zhang, Binbin Lu, Guojian Liu, Zhaoxia Wang

**Affiliations:** 1 Department of Oncology, The Second Affiliated Hospital of Nanjing Medical University, Nanjing, P.R. China; 2 Department of Oncology, The First Affiliated Hospital of Nanjing Medical University, Nanjing, P.R. China; 3 Department of Biochemistry and Molecular Biology, Nanjing Medical University, Nanjing, P.R. China; 4 Department of Medical Oncology, Nanjing Medical University Affiliated Cancer Hospital of Jiangsu Province, Cancer Institution of Jiangsu Province, Nanjing,P.R. China; 5 Department of Pathology, The Second Affiliated Hospital of Nanjing Medical University, Nanjing, P.R. China; H.Lee Moffitt Cancer Center & Research Institute, UNITED STATES

## Abstract

Long noncoding RNAs (lncRNAs) have been identified as oncogenes or tumor suppressors that are involved in tumorigenesis and chemotherapy drug resistance. Maternally expressed gene 3 (*MEG3*) is an imprinted gene located at 14q32 that encodes an lncRNA, and decreased *MEG3* expression plays an important role in multiple cancers. However, its biological role in the development of the chemoresistance phenotype of human lung adenocarcinoma (LAD) is unknown. This study aimed to observe the expression of *MEG3* in LAD and to evaluate its biological role and clinical significance in the resistance of LAD cells to cisplatin. *MEG3* expression was markedly decreased in cisplatin-resistant A549/DDP cells compared with parental A549 cells as shown by an lncRNA microarray. *MEG3* overexpression in A549/DDP cells increased their chemosensitivity to cisplatin both *in vitro* and *in vivo* by inhibiting cell proliferation and inducing apoptosis. By contrast, *MEG3* knockdown in A549 cells decreased the chemosensitivity. Moreover, *MEG3* was decreased in cisplatin-insensitive LAD tissues while p53 protein levels were decreased and Bcl-xl protein levels increased. Furthermore, patients with lower levels of *MEG3* expression showed worse responses to cisplatin-based chemotherapy. These findings demonstrate that *MEG3* is significantly downregulated in LAD and partially regulates the cisplatin resistance of LAD cells through the control of p53 and Bcl-xl expression. Thus, *MEG3* may represent a new marker of poor response to cisplatin and could be a potential therapeutic target for LAD chemotherapy.

## Introduction

Lung cancer is one of the most prevalent neoplasms worldwide, ranking as the first and second leading causes of cancer-related deaths in males and females, respectively. Non-small cell lung cancer (NSCLC) currently accounts for about 80% of all lung cancer cases [1, 2], and more than 65% of NSCLC patients present with locally advanced or metastatic disease [3]. Lung adenocarcinoma (LAD) is the most common histological subtype of NSCLC [4], but affected patients have limited access to early detection and timely treatment. Platinum-containing drugs such as cisplatin and carboplatin are typically used in LAD chemotherapy [5], although cisplatin resistance is the greatest obstacle of clinical LAD treatment.

Recent improvements in high-throughput gene expression analysis have led to the discovery that transcription from <2% of the human genome yields many short or long noncoding RNAs (lncRNAs) with limited or no protein-coding capacity because of a lack of open reading frames [6–9]. These show dysregulated expression in several human diseases but their exact biological functions are unclear. In our previous study, we reported that miR-224 could promote cisplatin resistance in A549/DDP cells via regulating G1/S transition and apoptosis by targeting p21^WAF1/CIP1^ [10]. The dysregulation of lncRNAs has also been shown to participate in tumorigenesis through promoting cellular proliferation and inducing apoptosis in multiple tumors. For example, lncRNA uc002mbe.2 expression was significantly downregulated in hepatocellular cancer (HCC), and its overexpression contributed to the trichostatin-induced apoptosis of HCC cells [11]. Similarly, lncRNA AK126698 was thought to regulate the chemoresistance of NSCLC cells through the Wnt signaling pathway [12], and our previous study found that lncRNA HOTAIR overexpression contributed to LAD cell cisplatin resistance via the regulation of p21 expression [13]. Moreover, in breast cancer patients with the single nucleotide polymorphism rs6983267 genotype, the novel lncRNA CCAT2 was demonstrated to reduce chemosensitivity to fluorouracil [14]. However, although some studies have elucidated the lncRNA functions in tumor chemoresistance, the underlying mechanisms are less well documented. An understanding of these mechanisms would improve LAD treatment and enable new targets for tumor chemotherapy to be identified.

LncRNA *MEG3* is a maternally expressed imprinted gene that is part of the *DLK1–MEG3* locus located on human chromosome 14q32 [15]. It is expressed in normal human tissues, especially in brain and the pituitary, and is thought to be a tumor suppressor [16]. Recent studies showed that *MEG3* expression is disrupted in various human cancers, such as bladder cancer, glioma, and HCC [17–19]. Moreover, Zhang et al. demonstrated that *MEG3* expression was reduced in meningioma compared with normal controls, and that this loss was associated with tumor grade [20]. Conversely, increased *MEG3* expression was reported to regulate NSCLC cell proliferation and apoptosis through the activation of p53 [21]. However, the functions and detailed mechanisms of *MEG3* in the cisplatin resistance of LAD remain elusive.

In this study, therefore, we investigated the role of *MEG3* in the chemosensitivity of LAD cells to cisplatin by analyzing its function both *in vitro* and *in vivo*. We demonstrated that *MEG3* expression was significantly decreased in cisplatin-resistant A549/DDP cells compared with that in parental cells, using lncRNA microarray and quantitative reverse transcriptase qRT-PCR. Ectopic expression of *MEG3* reduced A549/DDP cell cisplatin resistance, while *MEG3* knockdown increased this through regulation of cell proliferation and apoptosis. We further verified that overexpression of *MEG3* induced the mitochondrial apoptosis pathway via p53 and Bcl-xl activation. Our research confirms for the first time that, as a tumor suppressor, *MEG3* improves LAD chemosensitivity, and shows that it has potential to be used as a therapeutic target to reverse the cisplatin resistance of LAD patients.

## Materials and Methods

### Cell lines and culture

The human LAD cell line A549 was purchased from the Cancer Institute, Chinese Academy of Sciences. The cisplatin-resistant cell line A549/DDP was selected by continuous exposure to increasing concentrations of cisplatin followed by culturing in medium containing 1.0 μg/ml cisplatin to maintain the cisplatin resistance. All cell lines were cultured in RPMI 1640 medium (GIBCO-BRL, Grand Island, NY) supplemented with 10% fetal bovine serum, 100 U/ml penicillin, and 100 mg/ml streptomycin. They were grown under an atmosphere of 5% CO_2_ with humidity at 37°C. In all experiments, exponentially growing cells were used.

### Patients and tissue samples

Forty-one tumor tissues were obtained from advanced LAD patients who underwent cisplatin-based chemotherapy at the First or Second Affiliated Hospital of Nanjing Medical University between April 2007 and November 2009. All patients were histopathologically diagnosed with LAD and accepted first-line chemotherapy with cisplatin in combination with gemcitabine or paclitaxel for a maximum of four cycles. Tissue samples were divided into “sensitive” (complete or partial response, CR+PR; n = 20) and “insensitive” (stable or progressive disease, SD+PD; n = 21) groups according to the patients’ responses, as assessed by medical image analysis and detection of serum tumor markers after four cycles of cisplatin-based chemotherapy. All tissues were immediately frozen in liquid nitrogen and stored at −80°C until required for total RNA extraction. Tumor staging was determined according to the tumor–node–metastasis (TNM) classification of the International Union against Cancer. Written informed consent was provided by patients or their guardians. This study was approved by the Chinese Medical Association Society of Medicine’s Ethics Committee.

### RNA extraction and qRT-PCR

Total RNA was extracted from tissues or transfected cells using TRIzol reagent (Invitrogen, Carlsbad, CA). RT reactions were performed using *MEG3*-specific primers and a Takara Reverse Transcriptase kit (Takara, Dalian, China) according to the manufacturer’s instructions. The *MEG3* expression level was quantified using SYBR Green-based PCR (Takara) using *MEG3*-specific primers: forward, 5′-CTGCCCATCTACACCTCACG-3′; and reverse, 5′-CTCTCCGCCGTCTGCGCTAGGGGCT-3′, and normalized to the expression of *GAPDH*, which was amplified using specific primers: forward, 5′-GTCAACGGATTTGGTCTGTATT-3′; and reverse, 5′-AGTCTTCTGGGTGGCAGTGAT-3′.

### LncRNA microarray

Total RNA was isolated from cisplatin-resistant A549/DDP cells and parental A549 cells as described above, and the quality and quantity of the RNA samples were assessed. LncRNA microarray V2.0 analysis supported by CapitalBio Corporation (Beijing, China) was performed to detect lncRNAs and protein-coding mRNAs in the genome. LncRNAs were purified from total RNA after repeat sequences and ncRNAs shorter than 200 bp were deleted. In total, information about 30,756 lncRNAs was obtained from authoritative data sources including UCSC, RefSeq, H-Invitational Database, and other related literature, and bioinformatic analysis of microarray data was performed by CapitalBio Corporation.

### Transfection of LAD cells

*MEG3* was subcloned into the pCDNA 3.1 vector (Invitrogen, Shanghai, China), and this was purified using DNA Midiprep or Midiprep Kits (QIAGEN, Hilden, Germany). Exponentially growing A549/DDP cells were seeded into 6-well plates and transfected with pCDNA-*MEG3* vector or empty vector using Lipofectamine 2000 (Invitrogen) according to the manufacturer’s protocol. *MEG3* overexpression was confirmed by qRT-PCR and western blot analyses 48 h after transfection.

Three small interfering (si)RNAs targeting *MEG3* (si-*MEG3* I, II, and III) were also transfected into A549 cells, and the most stable was chosen (si-*MEG3* II). A549 cells were then transfected with this si-*MEG3* or scrambled negative control siRNA (si-NC), and *MEG3* expression was detected by qRT-PCR 48 h after transfection.

### Flow cytometric analysis of cell cycle or apoptosis

LAD cells for apoptotic analysis were double stained with Annexin V–FITC and propidium iodide 48 h after transfection and analyzed using a flow cytometer (FACScan; BD Biosciences, Shanghai, China) equipped with CellQuest software (BD Biosciences). Cells were classified as viable, dead, early apoptotic, or apoptotic. The percentage of early apoptotic cells was counted and compared between cells receiving different treatment. Cells for cell cycle analysis were stained with propidium iodide using the BD Cycletest Plus DNA Reagent Kit (BD Biosciences). The relative ratio of cells in G0/G1, S, or G2/M phase was counted and respectively compared with control groups. Each experiment was performed in triplicate.

### 3-(4,5-dimethylthiazol-2-yl)-2,5-diphenyltetrazolium bromide (MTT) assay and colon formation assay

The IC_50_ was determined by an MTT assay. Transfected cells (3.5 × 10^3^/well) were seeded in 96-well plates and maintained in standard growth medium. Overnight, cells were exposed to different concentrations (0, 1, 5, 10, 15, 20, 25, 30, 35, and 40 ìg/ml) of cisplatin. After incubation for 48 h, 0.5 mg/ml MTT was added and incubated for another 4 h. The medium was then replaced with 150 μl dimethyl sulfoxide (Sigma–Aldrich, St. Louis, MO) and vortexed for 10 min. Absorbance at 490 nm was then surveyed by an automatic microplate reader. Cell viability was assessed at 0, 24, 48, 72, and 96 h with and without cisplatin treatment. For the colony formation assay, transfected cells (0.5 × 10^3^/well) were seeded in 6-well plates. After 14 d, cells were fixed with methanol and stained with 0.1% crystal violet (Sigma–Aldrich) and the number of colonies was counted.

### Hoechst staining assay

Cells were cultured in 6-well plates and Hoechst 33342 (Sigma–Aldrich) was added to the culture medium for 10 min. Cells were then washed with PBS, and changes in nuclear morphology were detected by fluorescence microscopy using a 365 nm filter for Hoechst 33342. The percentage of Hoechst-positive nuclei per optical field (at least 50 fields) was counted to quantify Hoechst 33342 staining.

### Western blot assay

Cells were lysed using the mammalian protein extraction reagent RIPA (Beyotime, Shangahi, China), together with a protease inhibitor cocktail (Roche, Pleasanton, CA), and phenylmethylsulfonyl fluoride (Roche). Protein lysates were separated by 10% sodium dodecyl sulfate polyacrylamide gel electrophoresis, transferred to 0.22 μm nitrocellulose membranes (Sigma–Aldrich), and incubated with specific antibodies against p53, mouse double minute 2 homolog (MDM2), Bcl-2, and Bcl-xl (Cell Signaling Technology, Danvers, MA). An anti-GAPDH antibody was used as a control. Signals were visualized using the ECL chromogenic substrate and quantified by densitometry using Quantity One software (Bio-Rad, Berkeley, CA).

### *In vivo* chemosensitivity assay

Male athymic BALB/c nude mice aged 4 weeks were housed under specific pathogen-free conditions and manipulated according to protocols approved by the Shanghai Medical Experimental Animal Care Commission. Transfected A549/DDP cells (pCDNA-*MEG3* and empty vector) were harvested and resuspended at a concentration of 2.0 × 10^7^ cells/ml. Suspended cells (0.1 ml) were subcutaneously injected into a single side of the posterior flank of each mouse. Tumor growth was examined every 3 days, and tumor volume was calculated using the equation V = 0.5 × D × d^2^ (V, volume; D, longitudinal diameter; d, latitudinal diameter). When the average tumor size reached approximately 50 mm^3^, cisplatin was administered by intraperitoneal injection at a dose of 3 mg/kg, once every other day, for a total of three doses. Four weeks after the injection, mice were killed and the subcutaneous growth of each tumor was examined. Primary tumors underwent hematoxylin and eosin staining and immunostaining analysis for proliferating cell nuclear antigen, p53, and Bcl-XL protein expression. All procedures were performed in accordance with the Nanjing University Guide for the Care and Use of Laboratory Animals formulated by the National Society for Medical Research.

### Statistical analysis

All data are shown as means ± SE and were analyzed using Prism 5.0 software. Statistical analysis in the form of a Student’s *t*-test (two-tailed), one-way analysis of variance, and Mann–Whitney U test was performed using SPSS 16.0 software. *P* values less than 0.05 were considered significant.

## Results

### Involvement of *MEG3* expression in cisplatin-resistant LAD cells (A549/DDP) and parental LAD cells (A549)

**Fig 1:**
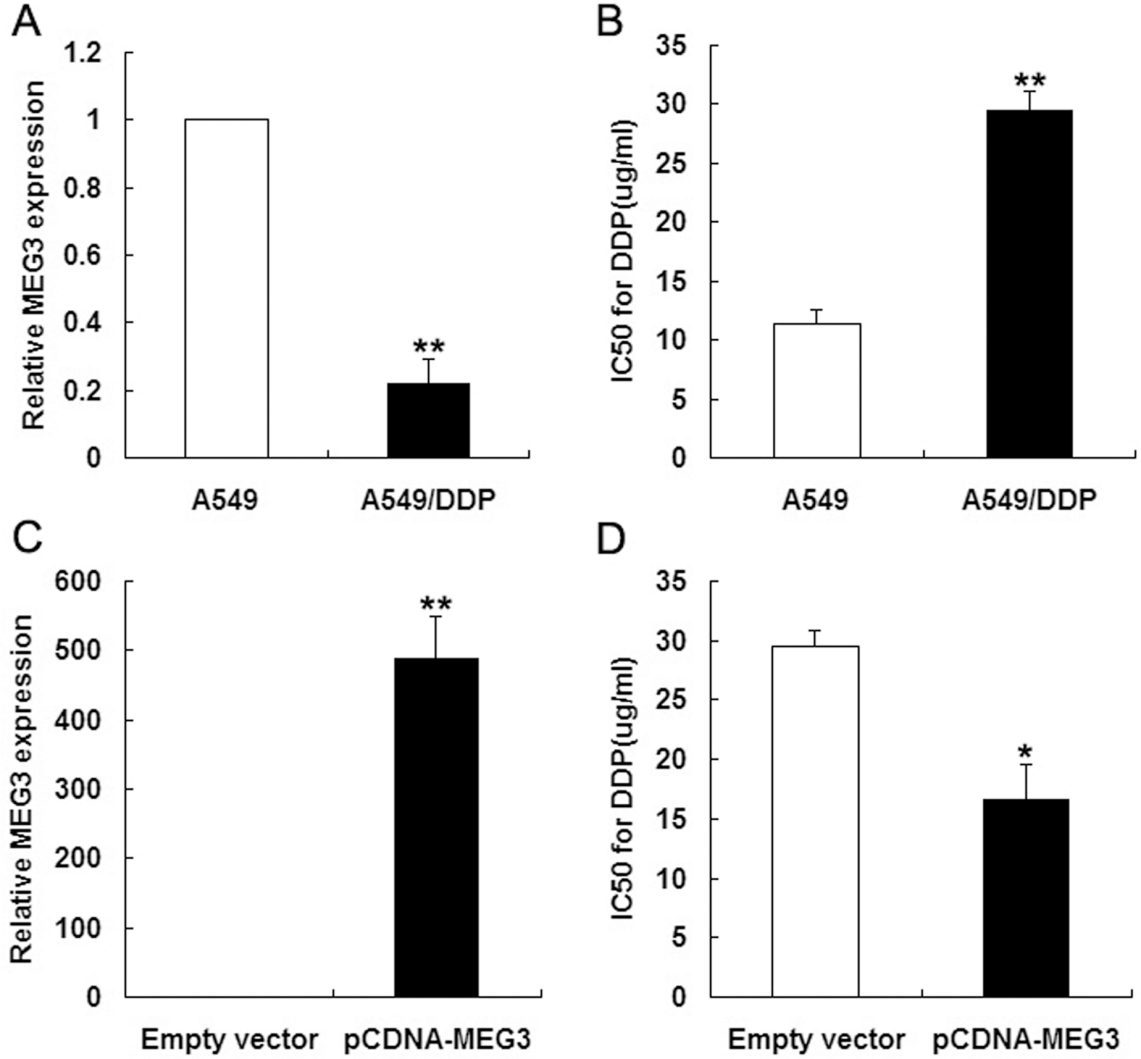
The level of *MEG3* expression level in LAD cells. (Fig 1A) qRT-PCR analysis of *MEG3* expression levels in A549 and A549/DDP cells. (Fig 1B) MTT assay of the IC_50_ values of A549 and A549/DDP cells to cisplatin. (Fig 1C) qRT-PCR analysis of *MEG3* expression levels following the transfection of A549/DDP cells with empty vector or pCDNA-*MEG3*. (Fig 1D) MTT assay of the IC_50_ values of empty vector- and pCDNA-*MEG3*-transfected A549/DDP cells to cisplatin. *P < 0.05, ** *P* < 0.01.

To identify the lncRNAs differentially expressed between cisplatin-resistant A549/DDP and parental A549 cells, we performed lncRNA microarray analysis. Of the 30,756 lncRNAs identified from databases, 664 showed differential expression between the cell types by more than 5-fold. Of these, 304 were downregulated and 360 were upregulated (Table 1), and *MEG3* showed the most downregulation in A549/DDP cells (by 61-fold). To validate the lncRNA microarray data, *MEG3* expression was determined in cisplatin-resistant A549/DDP and parental A549 cells by qRT-PCR and normalized to *GAPDH* levels. This found *MEG3* expression to be downregulated in A549/DDP cells by 4.5-fold compared with A549 cells (Fig 1A).

**Table 1:** Differentially expressed lncRNAs in A549/DDP cells compared with A549 cells as determined by microarray.

lncRNAs	Regulation	Ratio	lncRNAs	Regulation	Ratio
HIT000322758.11	Up	914.2004	ENST00000443252	Down	192.118
uc002llc.1	Up	250.9595	**MEG3**	**Down**	**61.61361**
uc002bbp.2	Up	141.9445	BX537613	Down	51.442579
uc003hsl.1	Up	135.3685	uc001yia.2	Down	48.626953
HIT000087455.12	Up	92.1891	14q(II-3)	Down	46.406529
ASO1629	Up	86.4334	NR_002766	Down	45.985177
ASO3765	Up	80.1581	LIT3318	Down	45.487044
NONE	Up	79.4499	NONE	Down	45.117236
uc003wyd.2	Up	72.9465	NR_033360	Down	42.646661
HIT000326960.4	Up	68.7971	uc010txc.1	Down	38.913981
uc001luz.1	Up	61.1428	AB074169	Down	35.9206
uc001uib.2	Up	53.4206	14q(II-2)	Down	35.540013
uc001lvd.2	Up	47.9377	uc010txe.1	Down	35.344072
NONE	Up	43.1377	NR_033358	Down	35.038002
BC032761	Up	27.0711	NONE	Down	33.132646
NR_003503	Up	26.0497	uc002sag.2	Down	31.316989
uc003kzj.2	Up	22.2284	AF070581	Down	31.148076
HIT000076908.10	Up	21.1488	NONE	Down	29.847659
ENST00000410370	Up	21.0256	NONE	Down	27.103947
uc002pec.2	Up	20.3612	NR_033359	Down	25.112085
uc004edk.1	Up	18.354	uc010txh.1	Down	24.008096
ASO3473	Up	17.2485	uc010txd.1	Down	22.722551
NR_036555	Up	17.1851	14q(II-21)	Down	21.993253
uc001uie.2	Up	16.2843	uc010txf.1	Down	21.907636

We previously reported differences in morphological characteristics and biological functions of A549/DDP and parental A549 cells [13]. In the present study, the IC_50_ of A549/DDP cells to cisplatin appeared to be higher than that of A549 cells (Fig 1B). We therefore explored the potential role of *MEG3* in the chemoresistance of LAD cells. *MEG3* was overexpressed in A549/DDP cells by transfecting them with pCDNA-*MEG3* for 48 h. qRT-PCR analysis revealed that *MEG3* expression was significantly increased by 487-fold in pCDNA-*MEG3*-transfected A549/DDP cells compared with A549/DDP cells transfected with empty vector (Fig 1C). Furthermore, an MTT assay showed that the IC_50_ of pCDNA-*MEG3*-transfected A549/DDP cells to cisplatin was significantly decreased compared with respective control cells (Fig 1D), indicating that the *MEG3* expression level of LAD cells affects their chemoresistance to cisplatin.

### *MEG3* overexpression inhibits A549/DDP cell apoptosis *in vitro*

**Fig 2:**
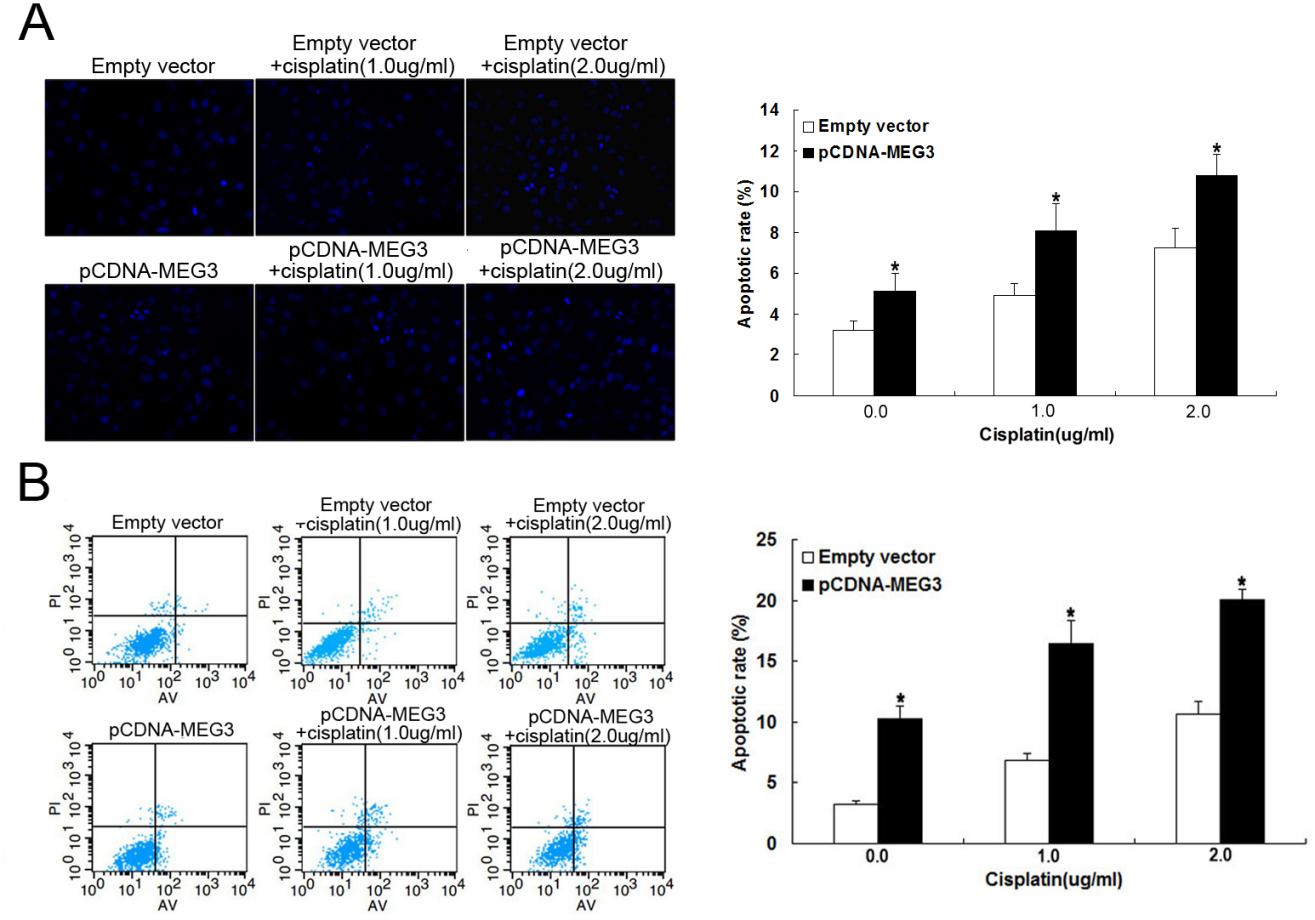
*MEG3* overexpression promotes A549/DDP cell apoptosis *in vitro*. (Fig 2A) Hoechst staining assay for cell apoptosis; the percentage of Hoechst-positive nuclei per optical field (at least 50 fields) was counted. (Fig 2B) Flow cytometry analysis of apoptosis in transfected A549/DDP cells in combination with increasing concentrations of cisplatin (0.0, 1.0, and 2.0 μg/ml). *P < 0.05.

Because high *MEG3* expression appeared to increase the chemosensitivity of A549/DDP cells to cisplatin, we further investigated the biological role and mechanism of *MEG3* in LAD cell chemoresistance. We used Hoechst staining and flow cytometric analysis to determine whether apoptosis was a contributing factor in chemoresistance. A549/DDP cells transfected with pCDNA-*MEG3* combined with cisplatin treatment showed a significantly increased rate of apoptosis with increasing doses of cisplatin (0.0, 1.0, and 2.0 ug/ml) compared with respective controls (Fig 2A). Thus, *MEG3* appeared to significantly increase cisplatin-induced apoptosis in cisplatin-resistant LAD cells. Flow cytometric analysis was also consistent with these findings. When treated with increasing doses of cisplatin (0.0, 1.0, and 2.0 ug/ml), the apoptotic rate of A549/DDP cells transfected with pCDNA-*MEG3* increased gradually compared with control cells transfected with empty vector (Fig 2B). Thus, *MEG3* reversed the cisplatin resistance of A549/DDP cells through the enhancement of apoptosis.

### *MEG3* overexpression impairs A549/DDP cell proliferation *in vitro*

**Fig 3:**
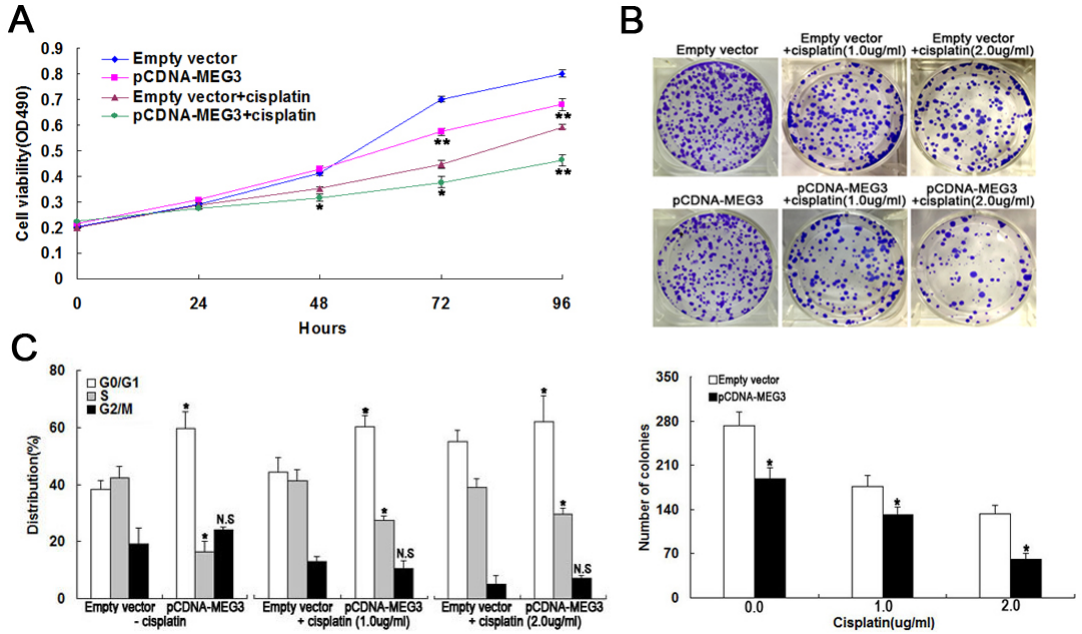
*MEG3* overexpression impairs A549/DDP cell proliferation *in vitro*. (Fig 3A) MTT assay of A549/DDP cell proliferation with or without cisplatin. (Fig 3B) Colony formation analysis of cell proliferation in combination with increasing concentrations of cisplatin (0.0, 1.0, and 2.0 μg/ml). (Fig 3C) Flow cytometry analysis of cell cycle distribution in combination with increasing concentrations of cisplatin. *P < 0.05, ** *P* < 0.01.

We next performed MTT and colony formation analysis to examine whether *MEG3* could affect cell proliferation *in vitro*. The MTT assay revealed that cell proliferation was impaired in A549/DDP cells transfected with pCDNA-*MEG3* combined with cisplatin treatment compared with control cells (Fig 3A). Similarly, colony formation analysis revealed that *MEG3* combined with increasing doses of cisplatin (0.0, 1.0, and 2.0 ug/ml) gradually decreased the number of colonies formed (Fig 3B).

Flow cytometric analysis showed that the percentage of pCDNA-*MEG3*-transfected A549/DDP cells in G1/G0 phase increased gradually and those in S phase decreased gradually with increasing doses of cisplatin compared with control cells (Fig 3C). Thus, the overexpression of *MEG3* also seemed to reverse the cisplatin resistance of LAD cells through the inhibition of proliferation.

### *MEG3* knockdown impairs A549 cell proliferation and apoptosis *in vitro*

**Fig 4:**
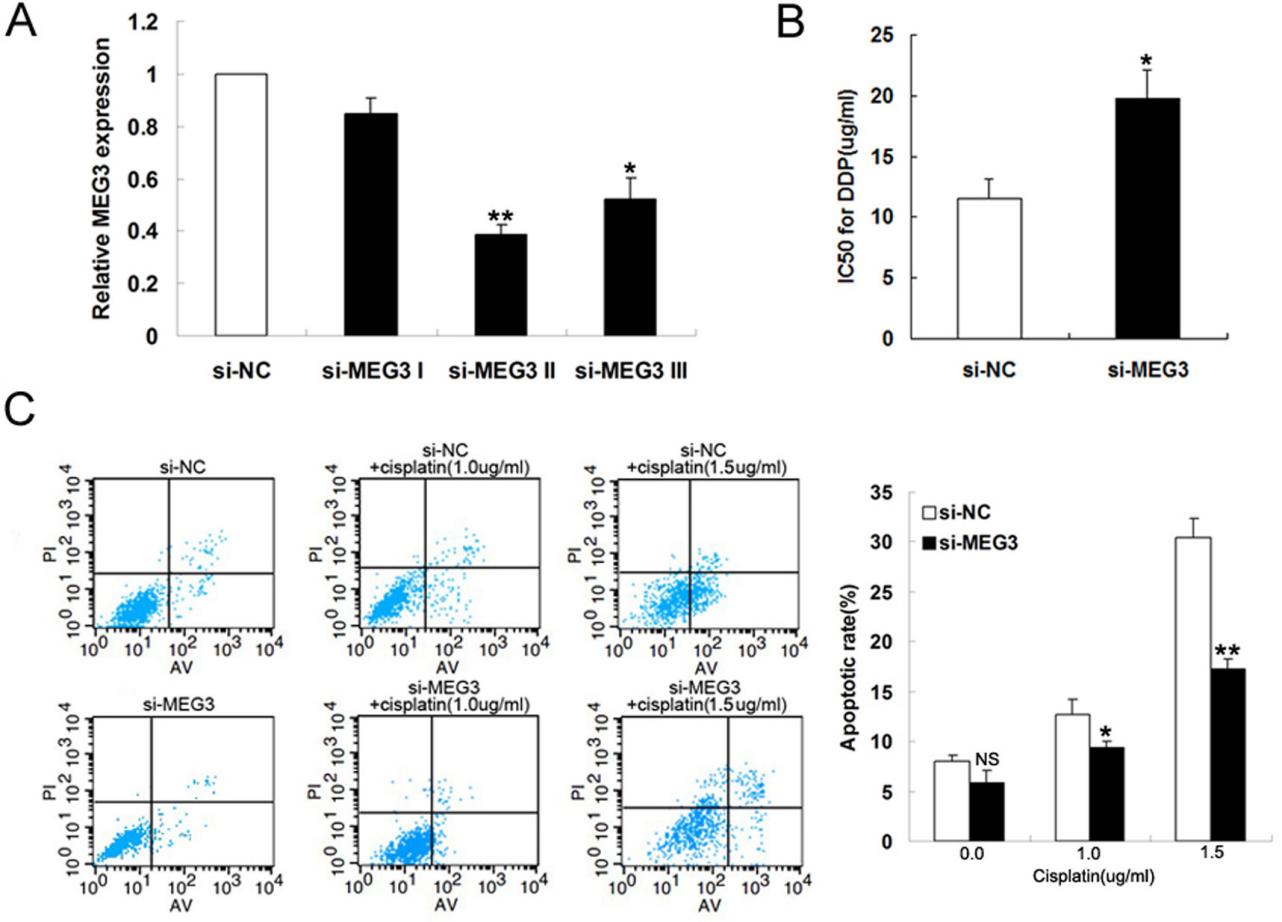
Knockdown of *MEG3* inhibits A549 cell apoptosis *in vitro*. (Fig 4A) qRT-PCR analysis of *MEG3* expression levels following the transfection of A549 cells with si-NC and si-*MEG3*. (Fig 4B) IC_50_ values of si-NC- and si-*MEG3*-transfected A549 cells to cisplatin. (Fig 4C) Flow cytometry analysis of apoptosis in si-NC- and si-*MEG3*-transfected A549 cells treated with increasing concentrations of cisplatin (0.0, 1.0, and 1.5 μg/ml). *P < 0.05, ** *P* < 0.01.

To further investigate the effect of *MEG3* on the cisplatin resistance of LAD cells, A549 cells were transfected with si-NC or siRNA-*MEG3* (si-*MEG3* I, si-*MEG3* II, and si-*MEG3* III). qRT-PCR showed that *MEG3* expression was significantly decreased in si-*MEG3* II-transfected cells compared with controls (Fig 4A). Moreover, *MEG3* knockdown significantly increased the IC_50_ of A549 cells to cisplatin, as demonstrated by the MTT assay (Fig 4B). Flow cytometry revealed that *MEG3* knockdown by si-*MEG3* gradually decreased the cisplatin-induced apoptosis of A549 cells treated with increasing doses of cisplatin compared with respective controls (Fig 4C).

**Fig 5:**
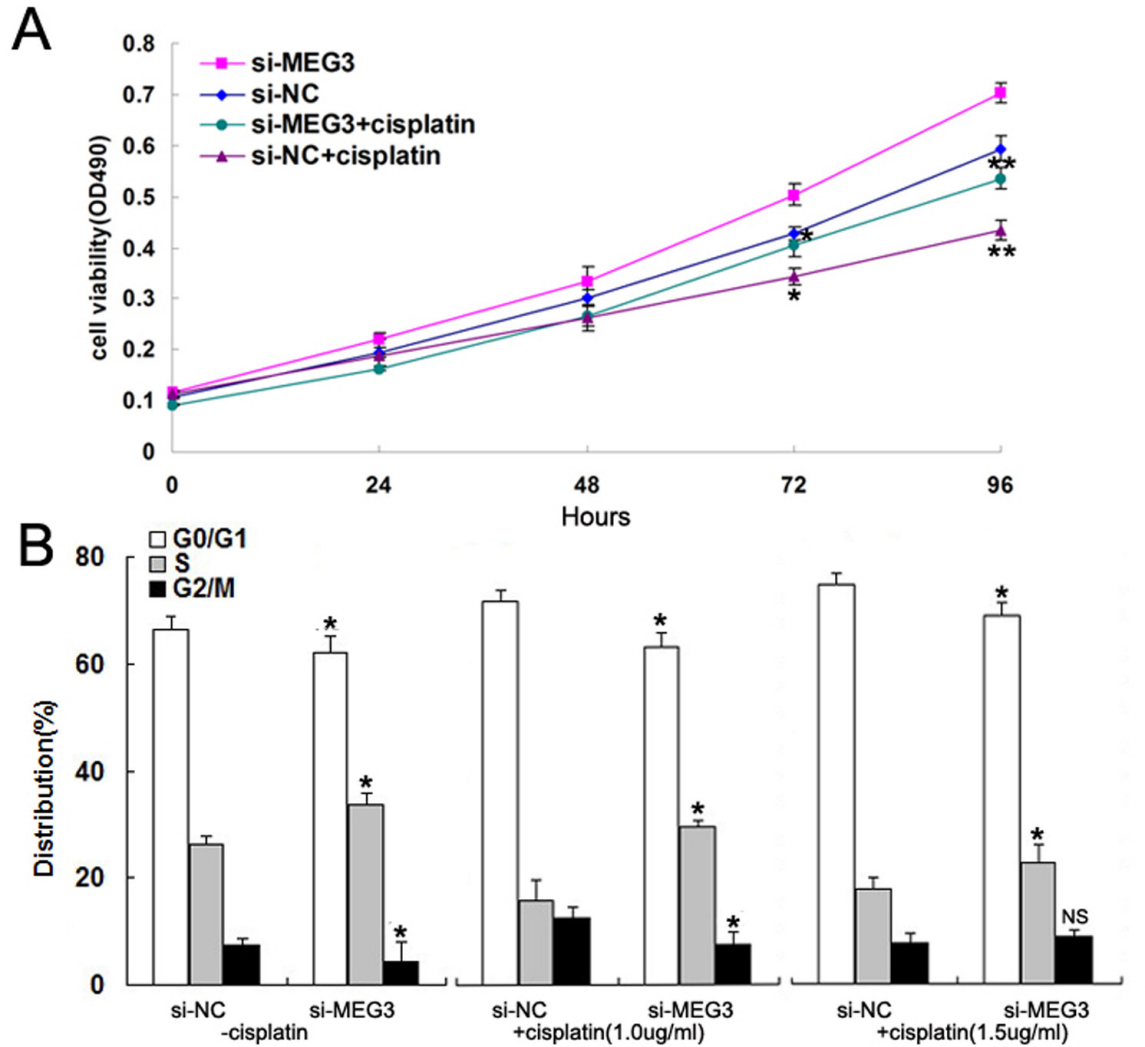
Knockdown of *MEG3* promotes A549 cell proliferation *in vitro*. (Fig 5A) MTT assay of the proliferation of si-NC- and si-*MEG3*-transfected A549 cells. (Fig 5B) Flow cytometry analysis of cell cycle distribution in combination with increasing concentrations of cisplatin (0.0, 1.0, and 1.5 μg/ml). *P < 0.05, ** *P* < 0.01.

Cell proliferation was also found to be promoted in si-*MEG3*-transfected A549 cells with or without cisplatin treatment compared with si-NC-transfected controls (Fig 5A). Flow cytometric analysis revealed that the percentage of cells in G1/G0 phase decreased gradually and those in S phase increased in si-*MEG3*-transfected A549 cells with increasing doses of cisplatin (0.0, 1.0, and 1.5 ug/ml) (Fig 5B). These results therefore suggest that *MEG3* knockdown increases the cisplatin resistance of A549 cells through apoptosis inhibition and the promotion of cell proliferation.

### *MEG3* induces the activation of p53 and Bcl-xl in LAD cells

**Fig 6:**
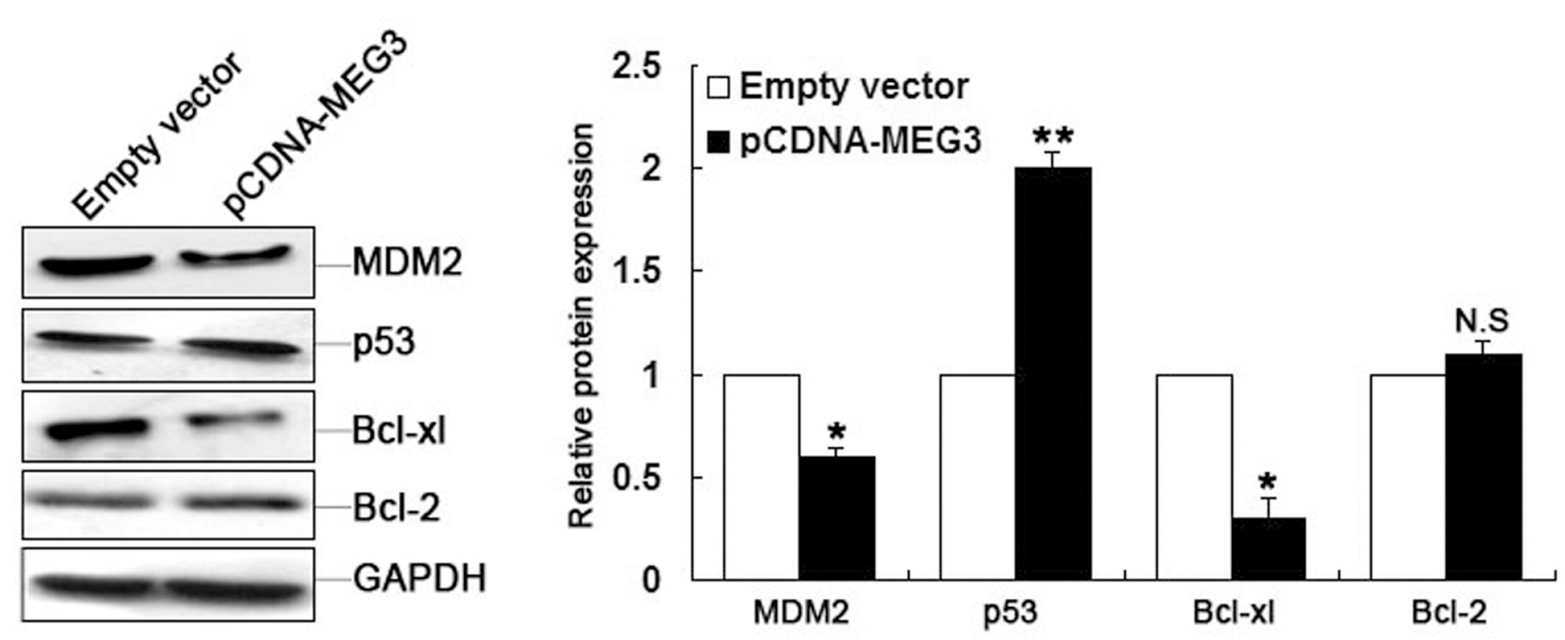
*MEG3* induces the activation of p53 and Bcl-xl proteins. Western blot analysis of MDM2, p53, Bcl-xl, and Bcl-2 expression levels after pCDNA-*MEG3* or empty vector transfection. *P < 0.05, ** *P* < 0.01.

To highlight the impact of *MEG3* in reversing the cisplatin resistance of A549/DDP cells through the enhancement of apoptosis, we performed western blotting to detect apoptotic signals in the form of MDM2 and Bcl-XL proteins 48 h after *MEG3* transfection combined with cisplatin treatment. This was because *MEG3* is thought to function as a tumor suppressor by inducing the activation of p53, which can translocate to the mitochondria during apoptosis, and MDM2 is responsive to p53 activation [22,23]. As shown in Fig 6, A549/DDP cells transfected with pCDNA-*MEG3* showed increased p53 protein and decreased MDM2 protein expression compared with controls. We next determined whether *MEG3* could increase apoptosis by regulating the mitochondrial apoptosis pathway. When *MEG3* was overexpressed, western blotting revealed decreased Bcl-xl expression, but no significant difference in Bcl-2 expression. These data further suggested that *MEG3*-induced apoptosis in LAD cell cisplatin resistance is induced by the mitochondrial apoptosis pathway involving p53 and Bcl-xl.

### *MEG3* improves the *in vivo* sensitivity of LAD cells to cisplatin

**Fig 7:**
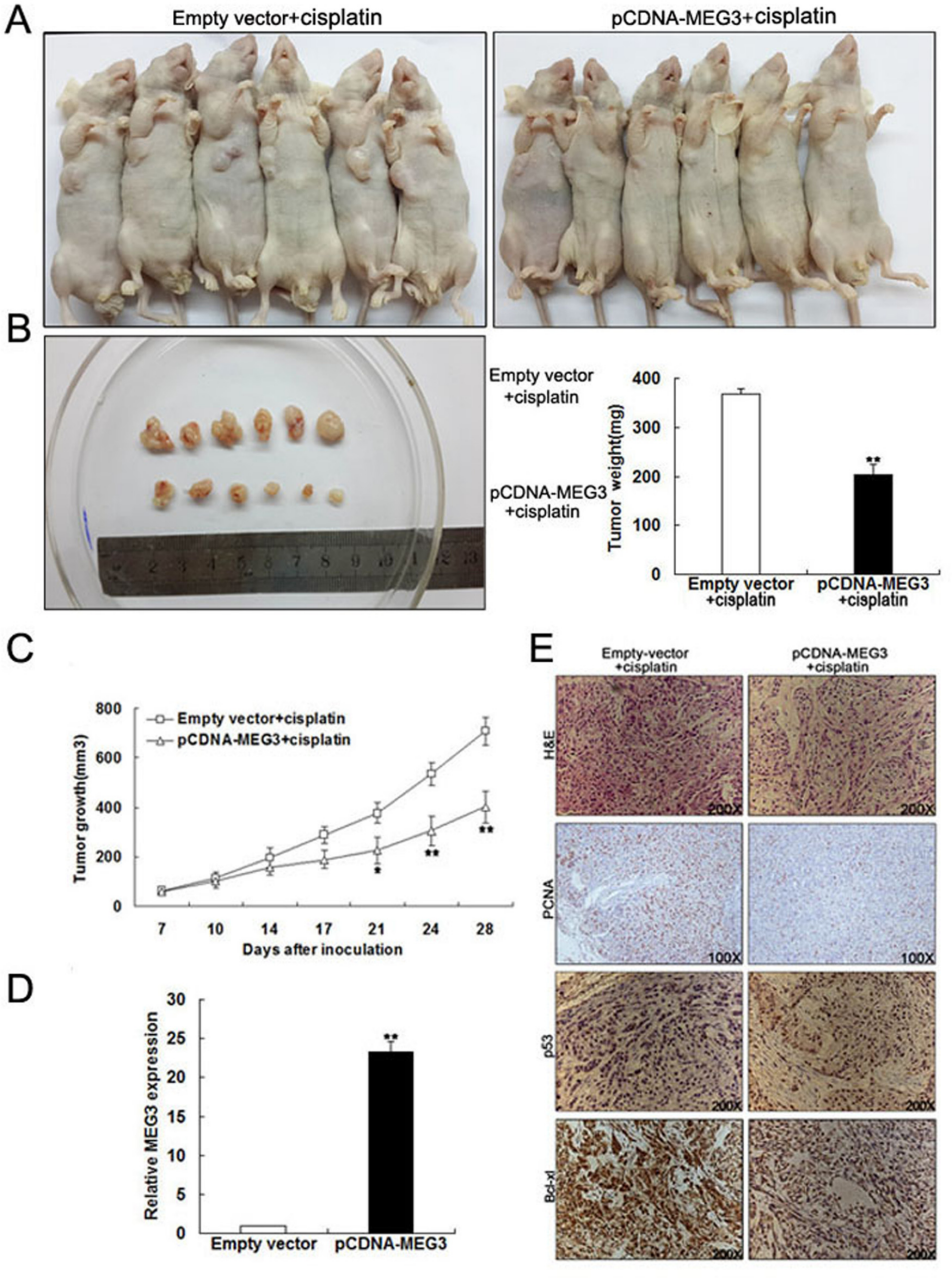
*MEG3* improves the *in vivo* sensitivity of A549/DDP cells to cisplatin. (Fig 7A, 7B) Four weeks after initial cisplatin administration, the tumor volume and average weight of mice receiving pCDNA-*MEG3*- or empty vector-transfected A549/DDP cells were recorded. (Fig 7C) Tumor volume was calculated twice weekly after cisplatin treatment. (Fig 7D) qRT-PCR analysis of *MEG3* expression levels in tumor tissues formed from pCDNA-*MEG3*- or empty vector-transfected A549/DDP cells. (Fig 7E) Tumors developed from pCDNA-*MEG3*-transfected A549/DDP cells showed lower PCNA protein levels, higher p53 protein expression, and lower Bcl-xl protein expression than those developed from control cells. Upper: H&E staining; second, third row and lower: immunostaining. Original magnification, ×100 or ×200. *P < 0.05, ** *P* < 0.01.

To further investigate the underlying roles of *MEG3* in enhancing the chemosensitivity of LAD cells to cisplatin, we used a nude mouse xenograft model. A549/DDP cells transfected with pCDNA-*MEG3* or empty vector were subcutaneously injected into mice, followed by treatment with cisplatin. Four weeks after the initial cisplatin administration, the volume and average weight of tumor xenografts was recorded (Fig 7A and 7B). As shown in Fig 7C, the tumors formed from pCDNA-*MEG3*-transfected A549/DDP cells grew significantly more slowly than those from controls following cisplatin treatment. It was also observed that the upregulation of *MEG3* led to the inhibition of tumor growth. qRT-PCR analysis of *MEG3* expression found it to be significantly higher in tumor tissues formed from pCDNA-*MEG3*-transfected A549/DDP cells than those from controls (Fig 7D). Additionally, immunostaining indicated that PCNA levels were lower in tumors formed from pCDNA-*MEG3*-transfected A549/DDP cells compared with tumors from control cells, while p53 protein levels were increased and Bcl-xl protein decreased, respectively, in these tumor types (Fig 7E). These results suggested that *MEG3* overexpression increased the *in vivo* chemosensitivity of LAD cells to cisplatin.

### *MEG3* correlates with the chemotherapy of LAD patients

**Fig 8:**
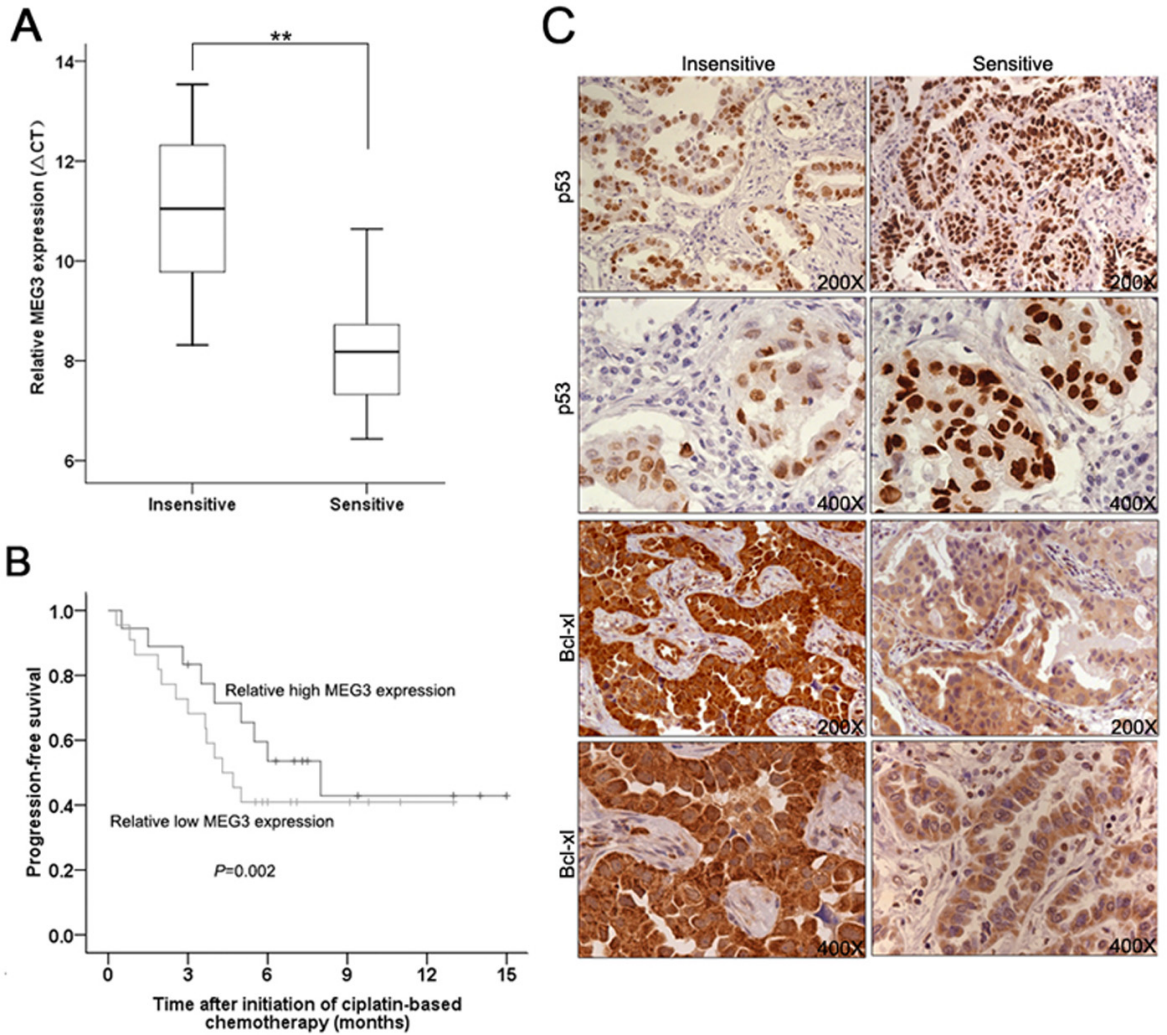
*MEG3* expression levels are positively correlated with the responses of patients to cisplatin-based chemotherapy. (Fig 8A) qRT-PCR analysis of *MEG3* expression levels in cisplatin-sensitive (n = 20) and cisplatin-insensitive (n = 21) LAD tissues. (Fig 8B) Kaplan–Meier survival analysis of the association between PFS of LAD patients and *MEG3* expression (log rank *P* < 0.001). (Fig 8C) Cisplatin-sensitive tissues exhibited higher p53 and lower Bcl-xl protein expression than cisplatin-insensitive tissues. Original magnification, ×200 or ×400. ** *P* < 0.01.

We next used qRT-PCR to investigate the *MEG3* expression of tumor tissues from 41 patients with advanced LAD treated with cisplatin-based chemotherapy. As shown in Fig 8A, the average *MEG3* expression level of ‘‘insensitive” tissues was significantly lower than ‘‘sensitive” tissues. Thus, the level of *MEG3* expression was strongly positively correlated with the response of patients to cisplatin-based chemotherapy. However, correlation analysis of MEG3 expression level of two groups with their clinical pathological features revealed no significant association (S1 Table). Kaplan–Meier survival analysis was performed to establish the association between *MEG3* expression and LAD patient progression-free survival (PFS). Those LAD patients with low *MEG3* levels (n = 17) had a shorter PFS than those with high *MEG3* levels (n = 24) (Fig 8B). Finally, we observed that p53 protein expression was increased and Bcl-xl protein expression decreased in ‘‘sensitive” tissues compared with ‘‘insensitive” tissues (Fig 8C). Together, these data strongly indicated that *MEG3* expression in advanced LAD patients is correlated with the response of patients to cisplatin-based chemotherapy.

## Discussion

As the most common subtype of NSCLC, LAD is attracting considerable attention particularly because its acquired resistance to drugs makes treatment difficult. Although the dysregulation of some lncRNAs is thought to affect LAD chemoresistance, the underlying molecular mechanisms of this have not been fully elucidated. Cisplatin is a commonly used chemotherapy drug and is the first chemotherapeutic choice for NSCLC. However, acquired drug resistance is a major obstacle to clinical chemotherapy.

Some mechanisms of cisplatin resistance have been reported, for instance, the Nrf2/ARE pathway in NSCLC and the calcium signaling pathway in ovarian cancer [24, 25]. Studies across a variety of cancer types demonstrate that misregulation of lncRNAs contributes to human disease or tumorigenesis, such as pituitary adenomas and gastric cancer [26–28]. Moreover, lncRNAs have been found to regulate a series of biological functions including genomic imprinting and transcriptional regulation. In this study, we found that the *MEG3* expression level was significantly decreased in cisplatin-resistant A549/DDP cells compared with parental A549 cells. This increased *MEG3* expression appeared to reverse the chemoresistance of A549/DDP cells to cisplatin. We found that lncRNA *MEG3* appeared to function as a tumor suppressor by regulating the p53 and Bcl-xl-induced mitochondrial apoptosis pathway, and showed that its increased expression was positively correlated with the sensitivity of LAD patients to cisplatin treatment.

Cell apoptosis is involved in the response of cancers to chemotherapy, radiation, or targeted therapy, and its induction may offer potential benefits to cancer therapeutics [29]. However, apoptosis is just one of the responses to cisplatin treatment. Wang et al. demonstrated that cisplatin exerted its cytotoxic effect partially through regulation of the p53 signaling pathway [30]. p53 is a DNA-binding tumor suppressor that plays a key role in many physiological processes, including DNA repair and apoptosis [31]. Furthermore, it can be induced by cisplatin to regulate cell cycle arrest [30]. MDM2 may inhibit the p53 transactivation function by engaging its amino-terminal transactivation domain [32], while the reactivation of p53 is a viable strategy for cancer treatment [33]. p53 is thought to act at multiple levels to induce mitochondrially-directed apoptosis [34], and triptolide was shown to be a potential chemotherapeutic agent for endometrial cancer via its actions on the p53-independent mitochondrial pathway [35].

Bcl-xl is a member of the Bcl-2 family of proteins that suppresses tumor apoptosis. This family is also reported to play an important role in mitochondrial permeabilization-induced apoptosis particularly through the p53–Bcl-xl interaction [36, 37]. In the present study, we showed that p53 expression was increased and that of Bcl-xl was decreased in *MEG3*-overexpressing LAD cells, while apoptosis was increased and the chemoresistance was partially reversed. We speculate that these observations reflect changes to the mitochondrial apoptosis pathway via p53 and Bcl-xl, but further work is needed to confirm this.

Taken together, our data demonstrated that the dysregulation of lncRNA *MEG3* underlies the cisplatin resistance of LAD, indicating that its overexpression may contribute to increased cisplatin chemosensitivity via the p53 and Bcl-xl induced mitochondria apoptosis pathway. This study enables us to better understand the functions of *MEG3* in LAD chemotherapy and suggest that *MEG3* could be used as a novel marker of poor response to cisplatin and a potential therapeutic target for LAD chemotherapy. Future study should investigate the molecular mechanisms underlying the functions of *MEG3* in the chemoresistance of LAD.

## Supporting Information

S1 Table Association between MEG3 level and clinicopathological features of LAD patients (sensitive e group and insensitive group). There was no significant association between pathological features and MEG3 expression level of LAD patient tissues.
(DOC)
