# Supplementary material for: The Long Noncoding RNA MEG3 Contributes to Cisplatin Resistance of Human Lung Adenocarcinoma
Source: PLoS One. 2015 May 20;10(5):e0114586. doi: 10.1371/journal.pone.0114586 (PMC4439130; doi:10.1371/journal.pone.0114586)
Supplement: S1 Table — There was no significant association between pathological features and MEG3 expression level of LAD patient tissues. (DOC) [file pone.0114586.s001.doc]

**Supporting Information Legends**

**S1 Table. Association between MEG3 level and clinicopathological features of LAD patients (sensitive group and insensitive group).** There was no significant association between pathological features and MEG3 expression level of LAD patient tissues.

| Factors | MEG3 level | | *P*-value |
| --- | --- | --- | --- |
|  | sensitive (n=20) | insensitive (n=21) |  |
| Sex |  |  | 0.395 |
| Male | 9 | 10 |  |
| Female | 11 | 11 |  |
| Age (years) |  |  | 0.268 |
| ≤60 | 8 | 9 |  |
| >60 | 12 | 12 |  |
| pathologic stage |  |  | 0.275 |
| I/II | 7 | 6 |  |
| III/IV | 13 | 15 |  |
| Tumor classification |  |  | 0.443 |
| T_1_/T_2_ | 10 | 12 |  |
| T_3_/T_4_ | 10 | 9 |  |
| Lymph node metastasis |  |  | 0.361 |
| N_0_ | 9 | 7 |  |
| N_1+2_ | 11 | 14 |  |
